# Evaluation of the Chemical Composition of Brazilian Commercial *Cymbopogon citratus* (D.C.) Stapf Samples

**DOI:** 10.3390/molecules13081864

**Published:** 2008-08-27

**Authors:** Luiz Cláudio Almeida Barbosa, Ulisses Alves Pereira, Ana Paula Martinazzo, Célia Regina Álvares Maltha, Róbson Ricardo Teixeira, Evandro de Castro Melo

**Affiliations:** 1 Department of Chemistry, Federal University of Viçosa, 36570-000, Viçosa, Minas Gerais, Brazil; E-mails: ulisses_ap@yahoo.com.br (U.A.P.); crmaltha@ufv.br (C.R.A.M.); teixeir5@yahoo.com.br (R.R.T.); 2 Department of Agricultural Engineering, Fluminense Federal University, Volta Redonda, Rio de Janeiro, Brazil; E-mail: a_martinazzo@hotmail.com (A.P.M.); 3 Department of Agricultural Engineering, Federal University of Viçosa, 36570-000, Viçosa, Minas Gerais, Brazil; E-mail: evandro@ufv.br (E.C.M.)

**Keywords:** *Cymbopogon citratus*, lemongrass, citronella grass, essential oil, losses and changes of oil

## Abstract

The concentration and the chemical composition of the essential oils obtained from different samples of *Cymbopogon citratus* were evaluated. Among the 12 samples investigated (11 dried leaf samples and fresh plant leaves), seven presented essential oil concentrations within the threshold established by the Brazilian legislation. The moisture content was also determined and the majority of the samples presented humidity contents near 12%. The GC and GC/MS analyses of the essential oils led to identification of 22 compounds, with neral and geranial as the two major components. The total percentage of these two compounds varied within the investigated sample oils from 40.7% to 75.4%. In addition, a considerable variation in the chemical composition of the analyzed samples was observed. The process of grinding the leaves significantly decreased (by up to 68%) the essential oil content, as well as the percentage of myrcene in the oils.

## Introduction

Brazil is recognized worldwide for its tremendous biodiversity, which can be explored in a rational way for the benefit of humankind. For example, various species from the Brazilian flora have been used in traditional medicine for the treatment of several illnesses. The level of knowledge associated with medicinal plants in Brazil is a legacy that stems from native tribes, European colonization and African cultural influence [1,2].

Despite its biodiversity, several plants currently used in the Brazilian folk medicine are not native species, but were brought to the country during the colonization process. Among such species is *Cymbopogon citratus* (DC.) Stapf, popularly known as citronella grass or lemongrass. This species belongs to the Poaceae family, which comprises approximately 500 genus and 8,000 herb species. The plant is a perennial grass that is widespread throughout the world, mainly in the tropical and savannah regions. The tea from its leaves has been widely used as an antiseptic, antifever, antidyspeptic, carminative, tranquilizer and stomachic. Several investigations have demonstrated the sedative, CNS depressor, analgesic, anti-microbial, and fungistatic activities of *C. citratus* leaves [3,4,5,6].

In 1998, the Brazilian government approved a legislation (RDC 519 of 06/26/1998) establishing the identity and quality of products made from plants to be used for infusions or decoctions. According to the regulation, dried leaves to be used for the preparation of *C. citratus* tea should have at least 0.5% (w/w) of essential oils and a maximum of 12% (w/w) moisture.

Considering the Brazilian legislation for moisture and essential oil contents, and the fact that *C. citratus* is largely consumed in Brazil and also exported for several countries, the present investigation was intended to evaluate the quality of some *C. citratus* dried leaf samples commercialized in Brazil. In addition, the utility of these commercial products for phytoterapeutic purposes is also outlined.

## Results and Discussion

### Quality of dried leaf samples regarding their moisture and essential oil contents

Eleven dried leaf samples of *C. citratus*, obtained from different locations in Brazil, were submitted to a volatile oil extraction by hydrodistillation in a Clevenger apparatus. A preliminary experiment was carried out in order to optimize the extraction time. For this, one sample was submitted to extraction during 30, 60, 90, 120, 150 and 180 minutes. We observed that 120 minutes was sufficient to extract all the oil present in the leaves and this time was used in all subsequent experiments.

The essential oil and moisture contents of the commercialized dried leaves are shown in Table 1. As previously mentioned, according to the Brazilian legislation, a commercial product made from *C. citratus* plants to be used in infusion preparations should have at least 0.5% (w/w) of essential oils, and the moisture contents cannot exceed 12% (w/w).

The moisture content is an important product quality parameter since high levels of humidity can facilitate enzymatic processes that can degrade the active principles. Moreover, the development of microorganisms under high-level moisture, which decreases the product therapeutic quality, is another issue to be taken into consideration [7,8]. As can be observed in Table 1, most of the commercial dried leaf samples presented moisture contents around 12% (w/w), in agreement with the Brazilian legislation. For comparison, the moisture content of the fresh plant leaves is also shown in Table 1.

**Table 1:** Average values of essential oil content in dried leaf products and fresh plant leaves of *Cymbopogon citratus*, commercialized in Brazil.

Sample	Sample origin	Moisture content (%)^*^	Oil Content (%, dried matter)^*^
**FP**	Viçosa, MG	78.37±0.60	0.76±0.10c
**1**	Tea bag, Viçosa, MG	11.11±0.10	0.23±0.02 e
**2**	Tea bag, Juiz de Fora, MG	10.52±0.67	0.20±0.03 e
**3**	Central Market, Belo Horizonte, MG	11.05±0.20	1.20±0.08 a
**4**	Central Market, Belo Horizonte, MG	10.35±0.07	0.70±0.01 c
**5**	Central Market, Belo Horizonte, MG	11.94±0.20	0.89±0.05 b
**6**	Central Market, Belo Horizonte, MG	11.41±0.03	0.98±0.09 b
**6g**	Central Market, Belo Horizonte, MG	12,39±0.19	0.45±0.04 d
**7**	Tea bag, Cascavel, PR	9.61±0.04	0.25±0.04 e
**8**	Tea bag, Cascavel, PR	9.76±0.21	0.22±0.04 e
**9**	Tea bag, Viçosa, MG	11.35±0.24	0.22±0.02 e
**10**	Central Market, Belo Horizonte, MG	12.40±0.26	1.00±0.04 b
**10g**	Central Market, Belo Horizonte, MG	11.74±0.13	0.60±0.03 c
**11**	Central Market, Belo Horizonte, MG	18.43±0.97	1.33±0.14 a
**11g**	Central Market, Belo Horizonte, MG	14.24±0.29	0.43±0.10 d

* Values are expressed as mean ± standard deviation (SD). Averages followed by the same letter do not differ among themselves, by Scott-Knott test, at 5% probability; FP= Fresh Plant; g = ground commercial samples.

The essential oil contents of samples **1**, **2**, **7**, **8** and **9** were almost half the minimum required by the legislation. Only samples **3**-**6**, **10** and **11** presented essential oil contents (ranging from 0.70% to 1.33%) higher than 0.5% (w/w). Once again, the percentage of essential oils obtained from the fresh plant leaves is included for comparison. It is important to note that the samples **3**-**6**, **10** and **11** are commercialized in the Central Market (Belo Horizonte, Minas Gerais state, Brazil) without any processing apart from drying, and the leaves are approximately 6 cm long. On the other hand, samples **1**, **2**, **7**-**9** were well known commercial brands, that are sold in small bags (10 g each) packed in a paper box, covered with a plastic film. In this case, the plants are ground and 90% of the material passes through a 0.5 mm sieve. In Table 1 these samples are identfied as named tea bags. Since the medicinal plant market in Brazil is not well regulated, it is difficult to trace the exact origin and conditions of cultivation of the various producers. So, for the samples analyzed in this work, only the region they were commercialized in is known.

It has been reported that the content of wood extractives decreases with storage time. This is associated with the degradation and oxidation of many compounds, especially unsaturated ones [9]. Although it is also known that for aromatic plants the essential oil content also decreases with the storage time [8], the expiration date of the tea bag samples had not been reached, so we suspected that the very low contents of essential oils of the samples **1**, **2**, **7**-**9** could be mainly attributed to the industrial grinding and drying processes. As the particles become smaller, the surface area increases and as a consequence the oil volatilizes faster. Moreover, the high grinding temperature can also lead to vaporization of volatile compounds [10]. As expected, the less polar and lower molecular weight compounds display higher losses. The packing materials such as cellulose or other synthetic polymers can also absorb volatile substances, reducing their content in the samples. Although all these factors could be associated with the reduced oil content of ground *C. citratus*, to evaluate the effect of the grinding process on the oil content, samples **6**, **10** and **11** were ground to pass a 1 mm sieve, resulting in samples **6g**, **10g**, and **11g**, respectively. These ground samples were immediately submitted to oil extraction. Table 1 shows that the oil contents of the ground samples were significantly reduced as follows: **6g** (from 0.98 to 0.45; 54% decrease), **10g** (from 1.00 to 0.60; 40% decrease), **11g** (from 1.33 to 0.43; 68% decrease). These results suggest that the volatile oil content of commercial dried leaf samples of *C. citratus* sold as tea bags had decreased in part due to the grinding process. Variations in the volatile oil contents related to the grinding process have been documented in the literature [10,11,12,13], but it should also be considered that the drying process could interfere with the content and composition of the oil in the final commercial product. As reported for *Lippia alba* [14] and *Mikania glomerata* [15] the air drying temperature, the sample size, the velocity of the drying air and the period of drying have influence on the amount and composition of the oil.

### Quantitative and qualitative analyses of essential oil components

The chemical composition of the essential oils obtained from *C. citratus* dried leaf samples and fresh plant leaves were studied by GC and GC/MS. The chromatographic and mass spectrometry analysis of the volatile oils resulted in the identification of 22 compounds, as presented in 2. A few other components present in some samples in trace amounts were also identified but they are not listed in the table as their presence was not considered of any relevance to the present investigation. The data presented in Table 2 shows variability in terms of qualitative and quantitative chemical composition. Although no detailed investigation on the origin of such differences were carried out, based on the literature they can be ascribed to several factors, including geobotanical conditions of the environment, cultivation method, plant age, photoperiod, harvest period, among others [16,17,18,19,20,21,22,23,24].

**Figure 1:**
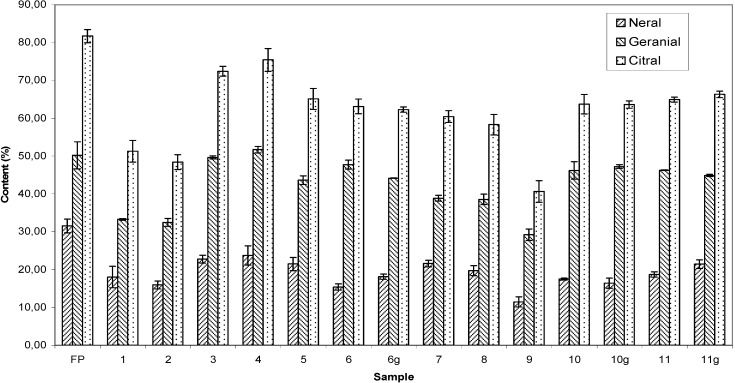
Citral content (%) in essential oil of dried leaf samples and fresh plant leaves of *Cymbopogon citratus*.

The investigated essential oils are composed mainly by citral, a name given to the mixture of the stereoisomers geranial and neral. As pointed out by Negrelle and Gomes [3], independently of their origin, *C. citratus* essential oils are composed of citral (30 to 93.74%), with general predominance of geranial. The variation of the citral content of the investigated dried leaf samples and fresh plant leaves is depicted in Figure 1. It was observed that among the dried leaf samples, the citral content ranged from 40.7% to 75.4% of the essential oil composition. The lower percentage of identified compounds for sample **9** (53.75%, Table 2), is mainly associated with the lower content of citral (40.61%, Table 2, Figure 1). As observed in the chromatogram (not shown) of this sample compared with the chromatogram of the oil from a fresh plant, several peaks with higher retention time were present. These were associated with degradation and oxidation products of citral that could not be identified. The infrared spectrum of sample **9** (not presented) also revealed a large and strong band centered at 3400 cm^-1^ and another absorption at 1716 cm^-1^ associated to vibrations of carboxyl group resultant of the oxidation of citral. In all samples, a preponderance of geranial over neral was also observed. The fresh plant leaves exhibited a citral content of 81.7%. According to the literature [25,26], in order for *C. citratus* essential oils to be considered as a high quality product, it must have citral content higher than 75%. Of the tested dried leaf materials, only sample **4** met this criterion.

It should be noted that the current Brazilian legislation (RDC 519 of 06/26/1998) does not establish minimum citral limits for the composition of dried leaves to be used for the preparation of *C. citratus* infusions or decoctions. On the other hand, according to the Brazilian Pharmacopeia, essential oils of *C. citratus* for phytotherapeutic purposes should contain at least 60% of citral [27]. As can be seen in Figure 1, the samples **3, 4, 5, 6, 6g, 7, 10, 10g, 11** and **11g** all fit in this criterion.

Regarding the chemical composition of the essential oils, besides citral, myrcene and geraniol stand out, since they were detected in amounts higher than 5% in some samples. As for the esters and alcohols identified in the essential oils of *C. citratus*, geraniol was by far the most frequently found one (1.5 to 10.4%), regardless of the plant origin [3].

**Figure 2:**
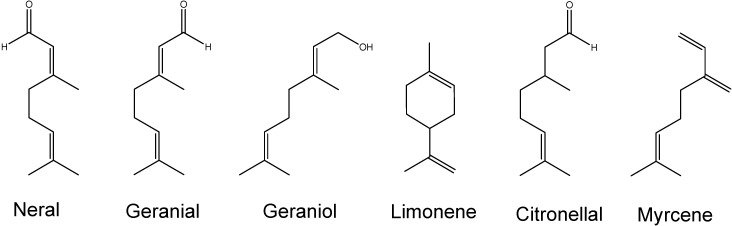
*C. citratus* markers.

Neral, geranial, limonene, citronellal, myrcene, and geraniol (Figure 2) were identified as maker compounds in the essential oils of *C. citratus*. Marker compounds are components or classes of chemical compounds responsible for the biological activity that can be used as references for quality control evaluation [26]. As shown in Table 2, neither limonene nor citronellal was found in any of the analyzed samples. The absence of the aforementioned commony used markers was also observed in *C. citratus* essential oils from Israel [28].

**Table 2:** Chemical composition of volatile oils obtained from *Cymbopogon citratus* (DC.) Stapf samples.

Peak No.	Component	RI	Relative peak area (%)^*^														
			FP	1	2	3	4	5	6	6g	7	8	9	10	10g	11	11g
1	6-methylhept-5-en-2-one	993	0.45	1.56	0.97	0.12	0.16	0.30	0.09	0.52	1.09	0.83	0.46	0.17	0.27	0.24	0.49
2	Myrcene	996	1.59	0.75	0.80	7.29	4.48	5.79	7.04	0.24	1.81	0.42	0.60	4.71	0.44	6.33	0.33
3	(*Z*)-*β*-ocimene	1039	-	-	-	0.19	0.14	-	-	-	-	-	-	-	-	0.13	-
4	*cis*-Linalool oxide	1081	-	0.04	-	-	0.18	-	-	-	-	-	-	0.12	0.22	0.14	0.08
5	*α*-Terpinolene	1090	-	1.32	1.57	0.80	0.83	1.44	-	-	1.77	-	0.51	-	0.90	0.81	1.13
6	Linalool	1091	0.81	-	0.12	-	-	-	0.66	0.15	-	1.64	-	-	0.06	-	-
7	Citronellol	1219	-	-	0.93	-	-	-	-	-	1.04	0.99	-	-	0.27	0.31	0.84
8	Neral	1235	31.53	18.02	15.94	22.76	23.72	21.48	15.38	18.11	21.61	19.73	11.44	17.49	16.41	18.67	21.45
9	Geraniol	1249	1.40	11.87	7.00	1.76	1.13	4.01	0.40	2.39	7.10	6.09	8.07	1.29	1.26	1.09	3.07
10	Geranial	1267	50.18	33.26	32.46	49.63	51.69	43.62	47.73	44.17	38.84	38.57	29.21	46.20	47.21	46.25	44.86
11	(*E*)-Anethole	1278	-	-	1.48	-	-	-	-	-	-	-	-	-	-	-	-
12	Undecan-2-one	1287	0.66	0.31	0.47	0.24	0.48	0.36	0.54	0.86	0.38	-	0.25	0.56	0.67	0.75	0.89
13	Geranyl acetate	1380	-	0.49	0.32	0.42	0.20	0.20	-	-	0.35	0.35	-	-	-	-	-
14	(*E*)-Caryophyllene	1417	-	-	1.00	-	-	-	-	-	-	-	-	-	-	0.06	0.67
15	*trans*-α-Begamotene	1435	-	0.16	0.22	-	-	-	-	0.12	0.15	0.15	0.17	-	0.07	-	-
16	(*E*)-β-Ionone	1484	-	0.24	-	-	-	-	-	-	-	-	-	-	-	-	0.14
17	Cuparene	1503	-	-	0.33	-	-	-	-	-	-	-	0.50	-	-	-	-
18	γ-Cadinene	1514	-	-	0.60	-	-	-	-	-	-	-	0.66	0.34	0.16	0.06	0.22
19	δ-Cadinene	1522	-	-	-	-	-	-	-	0.16	-	-	0.69	0.10	0.13	-	-
20	Caryophyllene oxide	1582	-	-	-	-	-	0.27	-	-	-	-	0.31	0.06	0.05	-	-
21	Torreyol	1643	-	-	0.72	-	-	-	-	-	-	-	0.77	-	-	-	-
22	Juniper camphor	1695	-	-	0.21	-	-	-	-	0.19	-	-	0.11	0.05	0.12	0.04	0.35
	Total identified:		86.62	68.02	65.14	83.21	83.01	77.47	71.84	66.91	74.14	68.77	53.75	71.09	68.24	74.88	74.52

^*^ Average of three repetitions. The variation coefficient was less than 10%. RI = Retention Index. g = ground commercial samples FP = Fresh Plant

**Figure 3:**
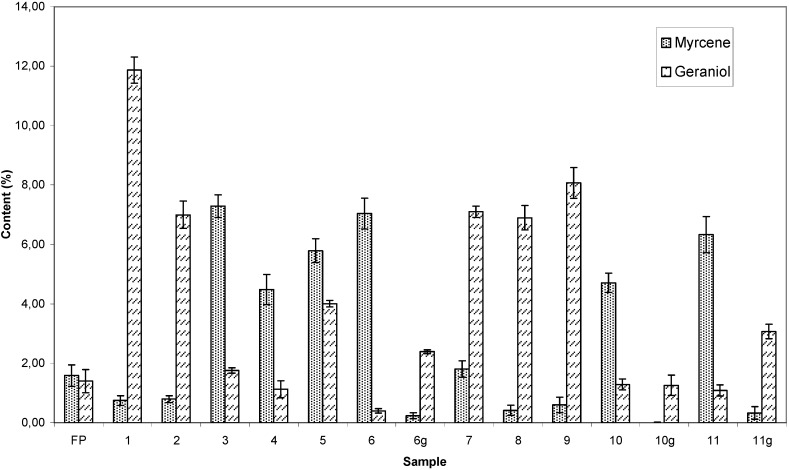
Variation in the myrcene and geraniol contents (%) in essential oil of dried leaf samples and fresh plant leaves of *Cymbopogon citratus*.

Figure 3 shows the variation of the myrcene and geranial contents in the essential oils of the investigated dried leaf samples and fresh plant leaves.

In the tea bag samples (**1, 2, 7, 8** and **9**) as well as in the ground samples (**6g**, **10g** and **11g**) the percentage of geraniol was higher than that of myrcene; a reverse trend was observed for the dried leaf samples commercialized at the Central Market in polyethylene bags (**3**, **4**, **5**, **6**, **10** and **11**). From the results, it is apparent that the grinding process also decreases the percentage of myrcene. The quantification of geraniol and myrcene can be considered important in view of properties associated with these metabolites. For example, geraniol prevents acute allograft [29] while myrcene displays analgesic properties [5].

## Conclusions

In summary, we have carried out an investigation on the essential oil chemical composition of eleven commercial samples of dried leaf and one fresh leaf sample of *Cymbopogon citratus*. Although a considerable chemical variability was observed, the essential oils were mainly composed of geranial and neral, with a preponderance of geranial. The oil concentrations of seven of the commercial samples met the standards established by the Brazilian legislation in terms of essential oil contents for samples to be used for infusion preparation. All of the tea bag samples presented essential oil concentrations below the legislation threshold (0.5% w/w), which might be a consequence of the industrial grinding and drying processes used. It was also determined that the grinding process led to a significant decrease of myrcene content in the dried leaf samples essential oils. Considering on the other hand the citral content required for phytoterapeutic purposes according to the Brazilian Pharmacopeia definition (60% w/w), most of the investigated samples met this criterion. Although the number of samples analyzed is small, the results obtained give an indication of the quality of some *C. citratus* commercialized in Brazil. In this aspect further studies should be carried out to provide the consumers with a broader knowledge of the quality of products available on the market.

## Experimental

### Plant material

The dried leaf commercial samples of *C. citratus* were divided into two groups according to their origin and processing for commercial purposes. Samples of the first group (**1, 2, 7, 8** and **9**) are commercialized in small bags (ground leaves, 10 g each) and produced by different Brazilian food companies. They were procured at supermarkets in Viçosa and Juiz de Fora (Minas Gerais State), and in Cascavel (Paraná State). Samples from the second group (**3, 4, 5, 6, 10** and **11**) were purchased at the Central Market, located in Belo Horizonte, Minas Gerais State. They were produced by small farmers in the Belo Horizonte region, and are sold as 6 cm long dried leaves, packaged in polyethylene bags, without a commercial brand. Samples **6**, **10** and **11** were ground in a knife-grinder (Tecnal, model TE-648, São Paulo, Brazil) to pass through a 1 mm sieve affording, respectively, samples **6g**, **10g,** and **11g**. Fresh *C. citratus* leaves (**FP**), measuring approximately 2 cm, were collected on the campus of the Federal University of Viçosa, Minas Gerais State, where a fresh *C. citratus* plant was cultivated in the Medicinal Herbs Garden of Plant Science Department at the Federal University of Viçosa, MG.

### Extraction and analysis of essential oil

Each sample (20 g) was subjected to extraction in a Clevenger apparatus for 2 hours, as described by Schaneberg and Khan [23]. All extractions were carried out in triplicate. The commercial tea bag samples were extracted without any previous preparation. The leaf samples obtained from the Central Market in Belo Horizonte were cut into pieces of approximately 1.5 cm length prior to extraction. The essential oils obtained were weighted and yields were determined in relation to the weight of dried samples. Leaf dry weight was calculated by drying each sample (2 g, held at 103 ± 2 ºC for 24 hours) according to a published method [30]. Each determination was carried out in triplicate.

### Gas chromatography-mass spectrometry (GC-MS)

Qualitative analyses were conducted with a GCMS-QP5050A system equipped with a mass selective detector (Shimadzu, Japan). The column was a DB-5 (J & W Scientific) fused silica column (30 m x 0.25 mm i.d., film thickness 0.25 μm). Column temperature was 40 ^o^C (2 min), increased at a rate of 3 ^o^C/min to 240 ^o^C, and kept at this temperature for 10 min. Injector temperature was 220 ^o^C. Helium was the carrier gas at a flow rate of 1.8 mL/min. An amount of 1 μL (1% w/v solution of the oil in dichloromethane) was injected and the split ratio was 1:10. The column pressure corresponded to 100 kPa. Mass detector conditions were as follows: temperature source 240 ºC; electron impact (EI) mode at 70 eV; scan rate 1 scan/s; mass acquisition range 29-450 u. The identification of the components was performed by comparison of their retention indexes (RI) [31], relative to a standard alkane series (C9-C24), and comparison of its mass spectrum with those on record in the Wiley library data base (Wiley 330000) or from literature [31]. GC analyses were carried out in triplicate, and accomplished with a GC-17A Series instrument (Shimadzu, Japan) equipped with a flame ionization detector (FID). Chromatographic conditions were as follows: fused silica capillary column (30 m x 0.22 mm) with a DB-5 bonded phase (0.25 μm film thickness); carrier gas, N_2_ at a flow rate of 1.8 mL/min; injector temperature 220 °C, detector temperature 240 °C; column temperature was programmed to start at 55 °C (isothermal for 2 min), with an increase of 3 °C/min, to 240 °C, isothermal at 240 °C for 15 minutes; injection of 1.0 μL (1% w/v in CH_2_Cl_2_); split ratio 1:10; column pressure of 115 kPa. Quantitative analyses were carried out in triplicate by external standardization for major constituents (Neral and Geranial 99%) within each oil sample. A calibration curve was prepared using solutions of each compound dissolved in dichloromethane in the following concentrations: 500, 1,000, 2,000, 3,000, 4,000, 5,000 ppm. Each solution was injected three times under the conditions previously described.
